# Cas6 specificity and CRISPR RNA loading in a complex CRISPR-Cas system

**DOI:** 10.1093/nar/gku308

**Published:** 2014-04-20

**Authors:** Richard D. Sokolowski, Shirley Graham, Malcolm F. White

**Affiliations:** Biomedical Sciences Research Complex, School of Biology, University of St Andrews, North Haugh, St Andrews, Fife KY16 9ST, UK

## Abstract

CRISPR-Cas is an adaptive prokaryotic immune system, providing protection against viruses and other mobile genetic elements. In type I and type III CRISPR-Cas systems, CRISPR RNA (crRNA) is generated by cleavage of a primary transcript by the Cas6 endonuclease and loaded into multisubunit surveillance/effector complexes, allowing homology-directed detection and cleavage of invading elements. Highly studied CRISPR-Cas systems such as those in *Escherichia coli* and *Pseudomonas aeruginosa* have a single Cas6 enzyme that is an integral subunit of the surveillance complex. By contrast, *Sulfolobus solfataricus* has a complex CRISPR-Cas system with three types of surveillance complexes (Cascade/type I-A, CSM/type III-A and CMR/type III-B), five Cas6 paralogues and two different CRISPR-repeat families (AB and CD). Here, we investigate the kinetic properties of two different Cas6 paralogues from *S. solfataricus*. The Cas6-1 subtype is specific for CD-family CRISPR repeats, generating crRNA by multiple turnover catalysis whilst Cas6-3 has a broader specificity and also processes a non-coding RNA with a CRISPR repeat-related sequence. Deep sequencing of crRNA in surveillance complexes reveals a biased distribution of spacers derived from AB and CD loci, suggesting functional coupling between Cas6 paralogues and their downstream effector complexes.

## INTRODUCTION

CRISPR-Cas (clustered regularly interspaced palindromic repeats—CRISPR associated) is a prokaryotic adaptive immune system found in many bacteria and almost all archaea. Small fragments of DNA (known as ‘spacers’) from invading genetic elements are captured in a poorly understood acquisition process and incorporated into the host genome, flanked by short tandem repeats, in a CRISPR locus. An upstream leader sequence harbouring a strong promoter ensures that the CRISPR locus is transcribed, generating pre-CRISPR RNA (pre-crRNA) that is in turn processed to generate mature crRNA by the Cas6 endonuclease (in type I and type III CRISPR-Cas systems) or host ribonucleases (in the type II system). Cleavage by Cas6 at two adjacent repeats generates a mature crRNA consisting of a single unique spacer flanked by repeat-derived sequences known as the 5′- and 3′-handles. crRNAs are incorporated into large multisubunit ribonucleoprotein complexes (known as effector, surveillance or interference complexes) and used to detect homologous sequences in non-self DNA or RNA, initiating a series of events that leads to the degradation of the invading genetic entity and therefore host immunity. Host DNA is rarely taken up (or actively discriminated against) by the CRISPR-Cas system, and the genomic CRISPR locus is protected against cleavage by effector complexes due to detection of a perfect match between the genomic locus and the CRISPR sequence flanking the spacer in the crRNA.

CRISPR effector complexes are classified into three main types (1). Class I, also known as Cascade, includes the signature subunit Cas3 and a crRNA-binding backbone composed of Cas7 subunits (2–4). The class III complexes share the Cas7-backbone with class I and have the signature subunit Cas10. Class III complexes can cleave either DNA (type III-A) or RNA (type III-B) targets and their cleavage mechanism is not yet well understood (5–8). By contrast, the class II complexes are comprised of a single large subunit known as Cas9 and generate crRNA using host ribonucleases and a unique genomically-encoded small RNA known as tracrRNA (9,10). Recently, the simplicity of the Cas9 system has led to its application in numerous experimental systems ranging from bacteria to human cells for the targeted cleavage of specific genomic regions, facilitating specific recombination or gene disruption (11).

**Figure 1.**
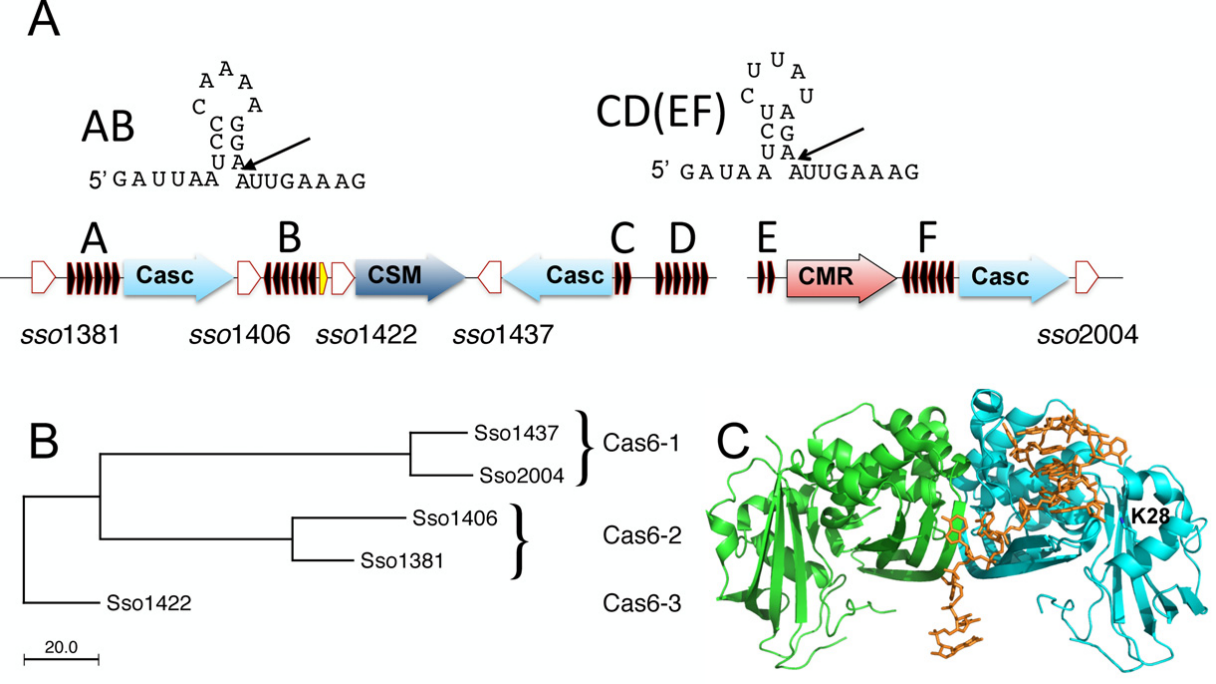
The CRISPR-Cas system of *Sulfolobus solfataricus*. (**A**) Schematic diagram showing CRISPR-Cas organization in the *S. solfataricus* P2 genome. Six repeat loci (small black arrows) comprising two distinct families (AB and CD) are present. Five Cas6 paralogues (white arrows) and five operons encoding effector complexes are indicated. The AB and CD families CRISPR repeats are shown in the hairpin conformation identified in the complex with Cas6-1 (15). The gene corresponding to ncRNA60 is shown next to *sso*1422. The CRISPR loci and cas genes are not generally contiguous and intervening genes are not indicated. (**B**) Midpoint-rooted neighbour joining phylogram of *S. solfataricus* Cas6 paralogues, which can be categorized into three families. (**C**) Structure of the Cas6-1 repeat RNA complex. The active site residue lysine 28 (K28) is indicated, positioning the active site at the base of the RNA hairpin.

CRISPR-Cas systems are particularly well represented in the archaea. The model organism *Sulfolobus solfataricus* encodes over 50 *cas* genes and six CRISPR loci (A–F) encompassing over 400 unique spacers (12,13) (Figure 1A). These repeats group into two different families (AB and CD) with distinct hairpin structures. Figure 1A shows the sequences of the AB and CD repeats in a hairpin conformation. Repeats E and F differ from the CD sequence by one and two nucleotide changes, respectively, but form the same hairpin stem and appear to be processed with similar kinetics by Cas6. Henceforth, they will be grouped under the CD family for simplicity. *Sulfolobus solfataricus* encodes three separate type I-A (Cascade) complexes, one type III-A (CSM complex) and at least one type III-B system (CMR complex). In addition, there are five clear Cas6 paralogues in the genome. Sso1437 and Sso2004 are 90% identical at the amino acid level and are hereafter referred to as the Cas6-1 family. This group has around 28% identity with the Cas6-2 family comprising Sso1381 and Sso1406. The Cas6-3 family has a single member, Sso1422, which shares ∼25% identity with Cas6-2 and 21% sequence identity with Cas6-1 (Figure 1B). *Sulfolobus solfataricus* Cas6-1 is a dimeric enzyme and lacks the canonical catalytic histidine sidechain found in the active site of most Cas6 orthologues (14). The structure of Cas6-1 bound to a CD-family repeat substrate revealed specific recognition of a short hairpin sequence in the repeat (Figure 1C), with cleavage at the base of the hairpin (15) as is seen in bacterial Cas6 enzymes (16,17). These hairpin structures are not expected to be highly populated for free pre-crRNA but appear important for substrate recognition by Cas6-1. By contrast, Cas6 from *Pyrococcus furiosus* is thought to wrap CRISPR RNA around the outside of the monomeric enzyme in a largely unstructured fashion (18). In *S. solfataricus* Cas6 enzymes have been shown not to co-purify with the CSM or CMR complexes (19,20), and to associate only weakly with the Cascade complex (21), suggesting that Cas6 is not an integral subunit of any of these systems.

In this study, we demonstrate that Cas6-1 from *S. solfataricus* has a strong preference for cleavage of the CD-repeat family and is capable of robust multiple turnover kinetics consistent with a role in generation of crRNA for multiple effector complexes. By contrast, the Sso1422 (hereafter Cas6-3) enzyme is less specific than Cas6-1 and is also responsible for the cleavage of a non-coding RNA (ncRNA60) precursor with a repeat-related sequence that is found in high levels in the CSM complex. Functional coupling of Cas6 enzymes and effector complexes in *S. solfataricus* is suggested by the biased distribution of crRNA found in the downstream effector complexes by next-generation sequencing.

## MATERIALS AND METHODS

### Cloning, gene expression and protein purification

Cas6-1 (Sso1437) was expressed and purified as described previously (14). The gene for Cas6-3 (*sso1422*) was amplified by PCR and cloned using NcoI and EcoRI sites into a derivative of the vector pEHisTEV (22), allowing expression with an N-terminal polyhistidine-tagged maltose binding protein (MBP) fusion. The K47A variant was constructed by site directed mutagenesis using the Quikchange protocol (Stratagene). The sequences of the PCR and mutagenesis oligonucleotides are available from the corresponding author on request. MBP-Cas6-3 protein was expressed in *Escherichia coli* C43 cells by induction at an O.D._600_ of 0.8 with 0.4 mM isopropyl β-D-1 thiogalactopyranoside (IPTG) and overnight incubation at 25°C with shaking. Cells were harvested by centrifugation, resuspended in buffer A (50 mM Tris–HCl pH 7.5, 500 mM NaCl, 30 mM imidazole, 10% glycerol), disrupted by sonication, centrifuged at 30 000 × g for 20 min and the supernatant filtered through a 0.2 μM filter. The cleared lysate was subjected to immobilized metal affinity chromatography on a 5 ml HisTrap FF column equilibrated with buffer A and eluted with a gradient of 30–500 mM imidazole in buffer A. Fractions containing MBP-Cas6-3 were pooled and applied to a Superdex 200 10/300 column equilibrated with buffer B (20 mM Tris–HCl pH 7.5, 300 mM NaCl, 10% glycerol) and eluted by isocratic flow in the same buffer. The final protein eluted in roughly equimolar quantities with the *E. coli* chaperone GroEL, suggesting problems with correct protein folding. The solubility of the protein was dependent on the presence of the MBP tag. These limitations meant that the MBP-Cas6-3 protein was only suitable for simple qualitative activity assays.

### Sequence and preparation of RNA substrates

RNA was purchased from IDT. The CRISPR repeats corresponding to *S. solfataricus* P2 CRISPR repeats of the C and D (CD) and A and B (AB) loci were synthesized in both fluorescently labelled (5′-fluorescein (FAM)) and unlabelled formats. An extra uracil nucleotide was added to the 5′ end of the repeat oligonucleotides to prevent fluorescence quenching by the guanine nucleotide that is the first *bona fide* nucleotide of both the AB and CD-repeat families. As a mimic of the Cas6 cleavage product, a fluorescently labelled CD sequence lacking the final eight 3′ nucleotides (CDproduct) was synthesized with a 3′-phosphate. The predicted Cas6 substrate leading to the generation of ncRNA60 was synthesized in an unlabelled format. The non-fluorescent oligonucleotides were end-labelled with γ-^32^P-ATP and polynucleotide kinase and purified as described previously (23)

AB: 5′-GAUUAAUCCCAAAAGGAAUUGAAAG

CD: 5′-GAUAAUCUCUUAUAGAAUUGAAAG

ncRNA60: 5′-UAAUGUGCCCCAAAAUGAAUUGAUAU

AB: 5′-[FAM]-UGAUUAAUCCCAAAAGGAAUUGAAAG

CD: 5′-[FAM]-UGAUAAUCUCUUAUAGAAUUGAAAG

CDproduct: 5’-[FAM]-UGAUAAUCUCUUAUAGA-P

### Single-turnover assays

Two micromolars purified recombinant Cas6 was incubated with 1–5 nM [γ-^32^P] ATP-labelled RNA (CD, AB or ncRNA60) at 60°C in nuclease reaction (NR) buffer (20 mM NaH_2_PO_4_/Na_2_HPO_4­_ pH 7.5, 100 mM NaCl, 5 mM EDTA, 0.5 mM dithiothreitol). This buffer supported a higher Cas6 activity than that reported previously (14). To stop the reaction, aliquots of 10 μl were quenched by addition to 30 μl acid phenol/chloroform (Ambion), vortexed briefly and centrifuged at 15 000 × g for 1 min. Five microlitres of the upper aqueous phase was removed and mixed 1:1 with formamide. Samples were heated at 95°C for 2 min and loaded onto a pre-run 20% denaturing polyacrylamide gel (20% acrylamide, 8 M urea, 1× Tris-borate-EDTA (TBE)) then electrophoresed at 80 W for 90 min in 1× TBE running buffer. Following electrophoresis, gels were scanned by phosphorimaging and analysed using Fuji Imagegauge software as described previously (24).

### Multiple-turnover assays

Cas6-1 (4–80 nM) was incubated with a large excess (4 μM) CD RNA in NR-buffer additionally supplemented with 2 μM bovine serum albumin (BSA). All other details were as described above.

### Electrophoretic mobility shift assay

A dilution series of Cas6-1 was prepared from 5 μM to 0.5 nM (monomer concentration) in NR buffer supplemented with 2 μM BSA, to which 0.5 nM [γ-^32^P] ATP-labelled RNA was introduced. After 15 min incubation at 20°C, reactions were gently mixed 1:1 with ficoll and 10 μl loaded under voltage (30 V) onto a non-denaturing­ 6% polyacrylamide, 1× TBE gel. Electrophoresis was completed at 120 V for 120 min in 1× TBE running buffer. Gels were phosphorimaged, quantified to determine the fraction of RNA bound and plotted using Kaleidagraph as detailed previously (23).

### Fluorescence quenching

Fluorescence quenching experiments utilized a Varian Cary Eclipse fluorimeter (*λ*_excitation_ = 480 nm) under temperature control at 25°C. In a 150 μl starting volume, changes to fluorescence intensity were recorded on addition of aliquots of Cas6-1 to 1 nM CD or CDproduct oligonucleotide with a 5′ fluorescein label. The emission data collected in the wavelength range 500–560 nm (1 nm data intervals) were summed, adjusted for dilution factor and normalized for baseline fluorescence. Assays were conducted in NR buffer supplemented with 2 μM BSA.

### Determination of Cas6 activity in size-fractionated lysates of *S. solfataricus*

Five grams *S. solfataricus* was grown, pelleted and lysed as described previously (19), resuspended in GF buffer (20 mM NaH_2_PO_4_/Na_2_HPO_4­_ pH 7.5, 250 mM NaCl, 5 mM ethylenediaminetetracetic acid (EDTA), 0.5 mM dithiothreitol (DTT), filtered through a 0.2 μm filter and applied to a Superdex 200 gel filtration column (GE Healthcare) equilibrated in GF buffer. Protein was eluted isocratically and 2 ml fractions were collected. Cas6 activity was determined as described above for single turnover conditions using a single time point of 60 min at 60°C. Aliquots were also analysed by western blotting using a polyclonal sheep antibodies raised against the Cas5–Cas7 (Sso1441–1442) complex of *S. solfataricus* Cascade, the Cmr7 (Sso1986) subunit of CMR and the Cas6-1 protein (SNBTS, Penicuik, Midlothian), using standard methods. In a separate experiment, recombinant Cas6-1 was passed through the same column under the same conditions in GF buffer.

## RESULTS

### *S. solfataricus* Cas6 paralogues have differing specificities for particular CRISPR-repeat sequences

**Figure 2.**
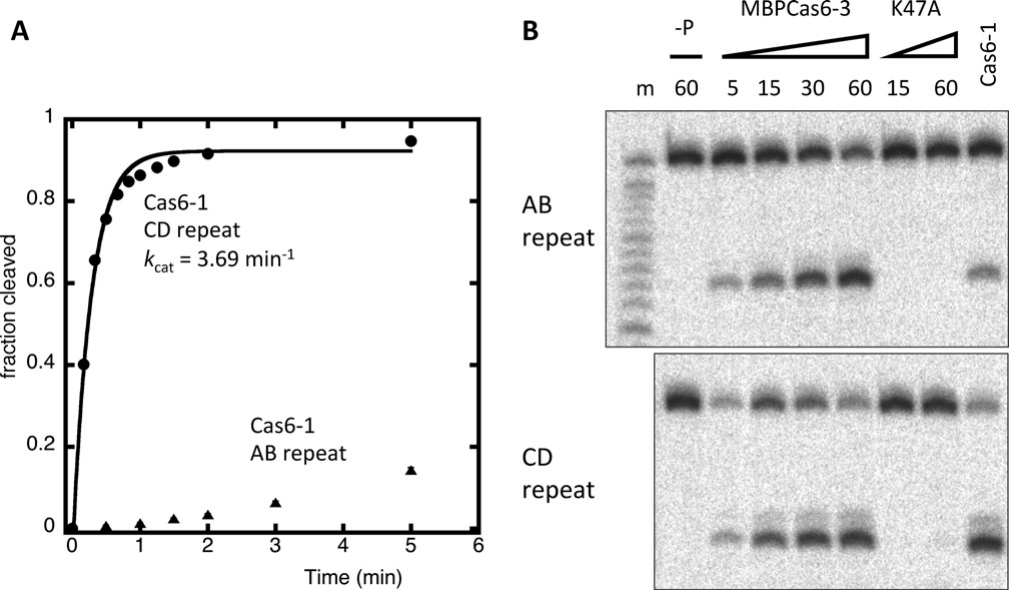
CRISPR-repeat specificity of Cas6-1 and Cas6-3. (**A**) Single turnover kinetic analysis of cleavage of the CD and AB families repeats by Cas6-1. The CD-repeat is cleaved with exponential kinetics and a rate constant of 3.69 min^−1^. The AB-repeat is cleaved much more slowly. Data points are the means of triplicate measurements and error bars showing the standard error are plotted although these are typically too small to see in the plot. (**B**) Denaturing gel electrophoresis monitoring cleavage of the AB and CD families repeats by the Cas6-3 endonuclease (MBP-Sso1422). Cleavage rates were roughly equivalent to one another and an active site variant protein (K47A) was inactive. Numbers show the time of incubation (in minutes). The control lacking protein is indicated as ‘−P’ and an alkaline hydrolysis ladder of the AB-repeat RNA is shown in lane ‘m’. Cleavage by Cas6-1 for 15 min for both these repeats is shown in the right hand lanes.

The co-existence of two families of CRISPR-repeat sequences and three families of Cas6 enzymes in the *S. solfataricus* genome raised the possibility that CRISPR repeats and *cas6* genes could have co-evolved. To test this possibility, we assayed the Cas6-1 enzyme against synthetic RNAs corresponding to the AB or CD family repeat sequences under single turnover kinetic conditions. The CD-repeat substrate was cleaved by Cas6-1 at a rate of 3.69 min^−1^, which is similar to the rate constants observed for other Cas6 enzymes (25). By contrast, the AB-repeat sequence was cleaved much (>50-fold) more slowly and did not show exponential single turnover kinetics (Figure 2A). In order to investigate the other Cas6 families we attempted heterologous expression of Sso1381, Sso1406 (Cas6-2) and Sso1422 (Cas6-3) in *E. coli*. We were unable to obtain soluble protein for Sso1381 or Sso1406 despite extensive attempts. Sso1422 (Cas6-3) could be obtained in soluble form when expressed as a fusion with MBP. We also generated and purified a K47A variant of Sso1422 that was designed to knock out a lysine residue predicted from sequence comparisons to be important for catalysis.

The MBP-Sso1422 fusion protein was obtained in sufficient amounts to allow initial investigation of its substrate specificity (Figure 2B). Cleavage of both the AB- and CD-family repeat sequences was observed under single turnover conditions with broadly similar rates. The K47A variant had no activity against either repeat, confirming that the activity observed for the wild-type enzyme was due to Cas6-3. These data suggest the Cas6-3 family is not biased towards one repeat sequence, unlike the Cas6-1 family. The site of cleavage could be mapped to the base of the short 3 bp hairpin stem of the AB-repeat (Figure 1), as expected for the generation of crRNA with an 8 nt 5′-handle.

### Cas6-1 binds the CD-repeat preferentially

**Figure 3.**
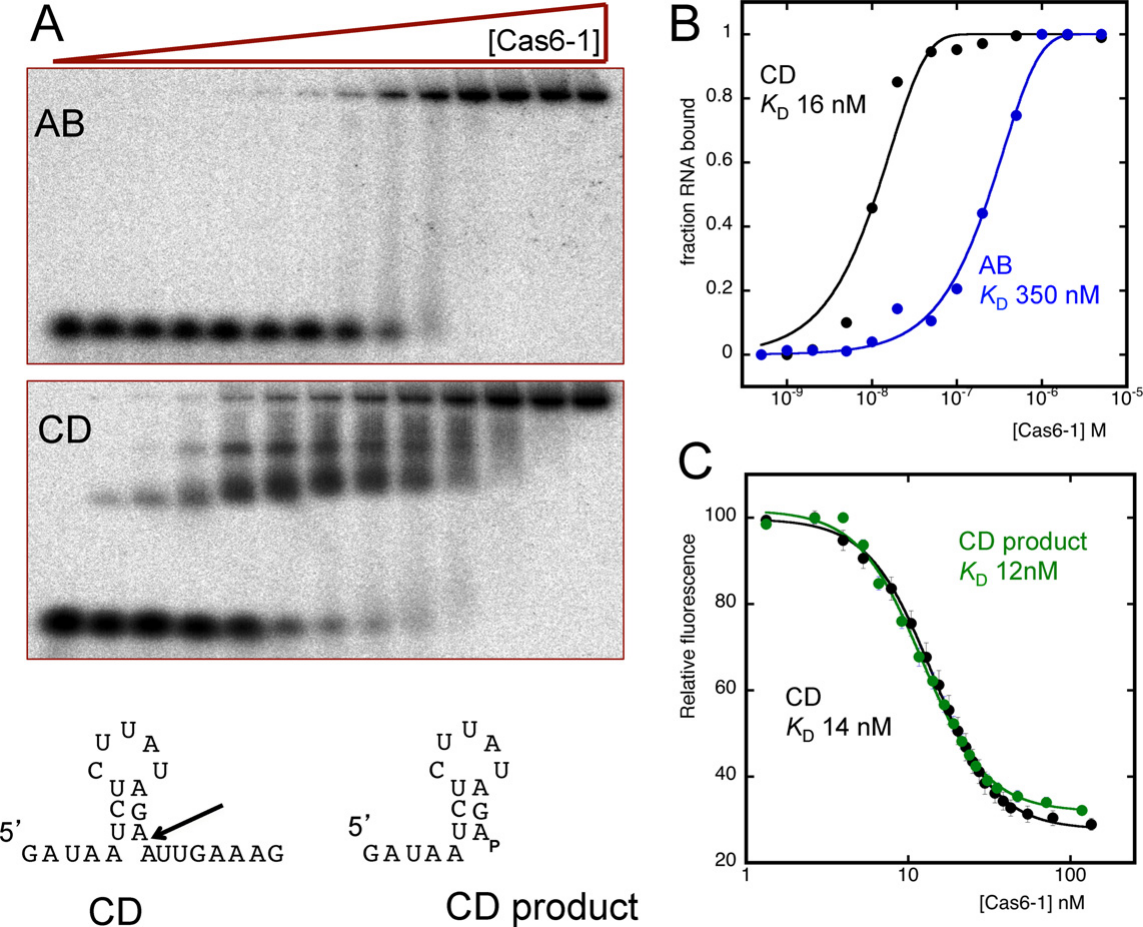
Repeat binding by Cas6-1. (**A**) Gel electrophoretic mobility shift assay showing Cas6-1 binding to 1 nM radioactively labelled AB- or CD-repeat at 20°C. (**B**) Quantification of the fraction of RNA bound for data in panel (A), fitted to a standard binding isotherm. (**C**) Cas6-1 binding to the CD and CD product RNA species monitored by fluorescence quenching of a 5′-fluorescein moiety at 25°C. Data points are means of triplicate measurements with SD. Standard error of the means (SEMs) shown. The data were fitted using a modified binding equation with a variable Hill coefficient to take apparent cooperativity into account (25).

An electrophoretic mobility shift assay (EMSA) was used to estimate the binding affinity of Cas6-1 for the CD and AB families repeats. The CD-repeat was bound with an apparent dissociation constant around 16 nM (Figure 3). This is >100-fold weaker than that observed for the Csy4 enzyme which has a *K*_D_ of 50 pM for its cognate substrate (25). Intermediate retarded species were observed, which may correspond to mixtures of one or two repeats binding to the dimeric Cas6 protein and potentially to cleaved repeats. In contrast, for the AB-repeat, the binding affinity was considerably lower (apparent *K*_D_ = 350 nM) (Figure 3) and no intermediate bands were observed. These experiments were carried out at 20°C, a temperature that minimizes cleavage of the RNA substrate by Cas6-1 as the enzyme has an optimal temperature for activity at 70°C or above (14).

To investigate the binding affinity in more detail, Cas6-1 was titrated against a fixed concentration of the CD-repeat sequence with a 5′-fluorescein label at 25°C. This experiment was also carried out using an RNA corresponding to the CD cleavage product of Cas6-1, with a 3′-phosphate at the point of cleavage mimicking the cyclic phosphate generated by Cas6. For both RNA species, progressive fluorescence quenching on titration of Cas6-1 was observed which could be fitted using a binding equation incorporating the Hill coefficient to take into account apparent cooperativity. Both the CD substrate and CDproduct were bound by Cas6-1 with an apparent dissociation constant around 14 nM, in good agreement with the value obtained from the EMSA experiment. Whilst we cannot rule out partial cleavage of the CD-repeat under these conditions, the most important conclusion arising from these experiments is the Cas6-1 binds its cleaved crRNA with a dissociation constant about 2.5 orders of magnitude higher than the other Cas6 enzymes that have been studied.

### Cas6-1 can operate under multiple turnover conditions to generate free crRNAs for effector complexes

**Figure 4.**
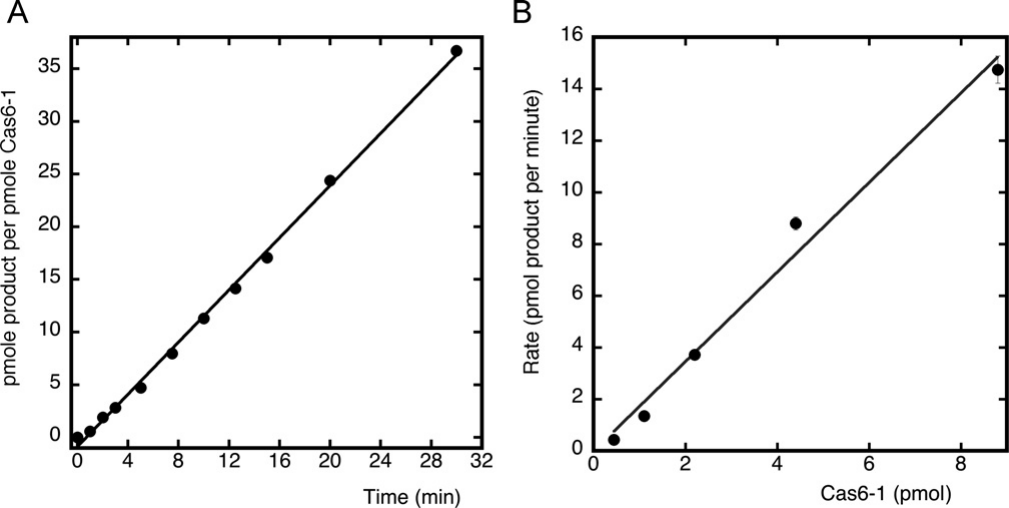
Multiple turnover cleavage of CRISPR repeats by Cas6-1. (**A**) 10 nM Cas6-1 was incubated with 4 μM ^32^P-labelled CD-repeat under multiple turnover conditions (see ‘Materials and Methods’ section). Over 35 turnovers were observed in the 30 min assay. (**B**) Secondary plot showing that, under multiple turnover conditions, the rate of product formation was linearly dependent on the enzyme concentration with a rate constant of 1.75 min^−1^. Error bars represent the standard errors of the rates obtained from linear curve fits as shown in (A).

All Cas6 enzymes studied to date have been analysed under single turnover conditions where the enzyme is present in large excess compared with the RNA substrate. Several Cas6 enzymes from different sources have been shown to catalyse only a single turnover, remaining bound tightly to the cleaved crRNA product. Biologically, this makes sense where a Cas6 enzyme is dedicated to the generation of crRNA for one effector complex, particularly where Cas6 is an integral component of the complex. However, the situation in *S. solfataricus* is more complex, with five Cas6 paralogues and at least five surveillance complexes. Accordingly, we investigated the ability of Cas6-1 to catalyse multiple turnover kinetics, a reaction cycle that requires the release of crRNA product after cleavage. When 10 nM Cas6-1 was incubated with a 400-fold excess (4 μM) of CRISPR-repeat substrate, multiple turnover was observed, with over 35 turnovers in 30 min (Figure 4A). The multiple turnover reaction rate varied linearly with enzyme concentration over a broad range of Cas6 concentrations (Figure 4B). The rate constant for this reaction was estimated as 1.75 min^−1^, which may be close to the *V*_max_ of the enzyme because substrate concentrations were much higher than the dissociation constant of ∼15 nM. Compared with a single turnover rate constant of 3.7 min^−1^ under equivalent conditions, this suggests that the overall reaction rate may be partially limited by substrate binding or product dissociation rather than the rate for the chemical step of catalysis, but the difference in rates was not large. Thus, it appears Cas6-1 is capable of generating free crRNA products in multiple turnover mode for incorporation into multiple distinct surveillance complexes.

### Cas6-1 activity in *S. solfataricus* extracts

**Figure 5.**
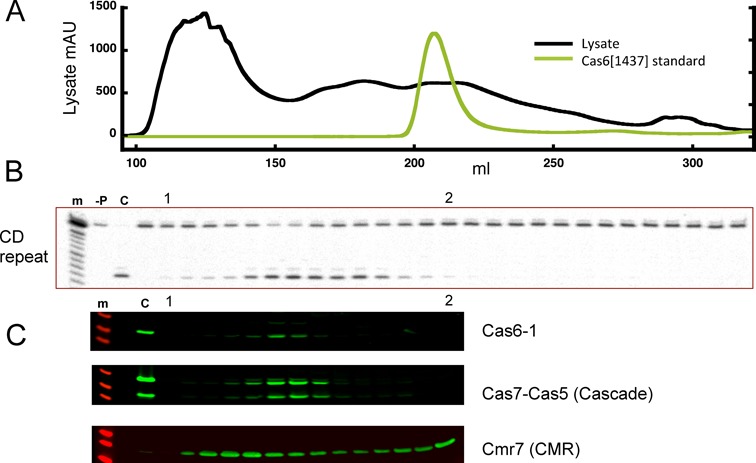
Cas6-1 activity in *Sulfolobus solfataricus*. *(***A**) UV absorbance traces showing total protein elution following application of an *S. solfataricus* cell lysate to a gel filtration column (black), and the elution profile of purified dimeric Cas6-1 (pale). (**B**) Nuclease activity against the CD-repeat in the cell extract. The gels are aligned with the UV traces shown in (A). Lane m: alkaline hydrolysis ladder; −P: no protein control; C: RNA digested with recombinant Cas6-1. (**C**) Western blots showing the coincident elution positions of Cas6-1 and the Cascade core subunits Cas7–Cas5. The elution position of Cmr7, a subunit of the CMR complex, is also shown. Lane m: protein markers; C: recombinant proteins; labels 1 and 2 serve to indicate identical fractions in panels (B) and (C).

To investigate Cas6 activity in *S. solfataricus* extracts, we lysed 5 g *S. solfataricus* biomass and passed it over a gel filtration column to fractionate proteins by size. Fractions were then assayed for Cas6 activity and analysed by western blotting with polyclonal antibodies raised against Cas6-1, the Cmr7 subunit of the CMR complex and the Cas5–Cas7 complex that forms the core of Cascade. The UV-absorbance trace showed proteins and other UV-absorbing material eluting in a complex pattern at retention volumes between 100 and 300 ml (Figure 5A). Recombinant dimeric Cas6-1, which has a native molecular weight of 65 kDa, elutes from the column in a single peak with a retention time centred on 210 ml. CD-repeat cleavage activity corresponding to Cas6-1 was detected in one peak eluting with retention volumes between 150 and 180 ml (Figure 5B). This activity eluted with a considerably smaller retention volume than recombinant Cas6-1, suggesting that the Cas6 activity is part of a larger complex. This was confirmed by western blotting using antibodies raised against Cas6-1, which showed that the protein eluted in one peak at the position corresponding to the peak of activity against the CD-repeat (Figure 5C). This is consistent with the hypothesis that the Cas6-1 enzymes are responsible for cleavage of the majority of CD-repeat sequences *in vivo*. Duplicate membranes were probed using antibodies raised against the heteromeric protein Sso1441–1442 (Cas7–Cas5, representing the core of the archaeal Cascade complex (21)). Cas6-1 clearly elutes co-incidently with Cascade, suggesting that they may form a complex in cell extracts, as suggested previously (21). We cannot rule out the possibility that co-incident elution is due to the interaction of Cas6-1 with other factors such as pre-crRNA or with other CRISPR-Cas complexes in cell extracts. A stable interaction with Cas6 was not observed in studies of the *Thermoproteus tenax* type I-A Cascade complex (26). However, antibodies raised against the Cmr7 subunit of the CMR complex show a peak eluting before Cascade (consistent with the large size of the CMR complex) and another peak corresponding to the dimeric Cmr7 protein (Figure 5C). These data suggest that Cas6-1 is not co-eluting with the CMR complex, consistent with earlier observations (19).

### A non-CRISPR RNA is abundant in the CSM complex and is processed by Cas6-3 *in vitro*

**Figure 6.**
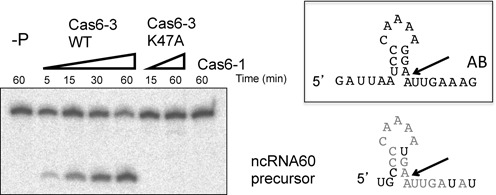
A non-CRISPR-repeat substrate for Cas6-3. ncRNA60 precursor is cut by Cas6-3 at a similar rate and site to the AB-repeat, but not by Cas6-1. The active site variant of Cas6-3 (K47A) was inactive. Lane −P indicates a no-protein control incubated for 60 min. Sequences and potential structures of the AB-repeat and ncRNA60 precursor RNAs are shown on the right. Arrows show the positions cleaved by Cas6-3. For ncRNA60, the residues in grey match corresponding residues in the AB-repeat. Note the lack of potential to form a base paired hairpin stem.

Evidence supporting the functional coupling of Cas6-3 and CSM comes from the identification of a non-crRNA present in nearly 1.5% of the total sequence reads in the CSM complex as judged by next generation sequencing (20). By contrast, this species constitutes only 0.07% of the total RNA isolated from the CMR complex (19). The RNA, previously annotated as ncRNA60 by Sorek *et al.* (27), has no known function and is found adjacent to the *sso1421* gene (and therefore very close to the *sso1422* gene encoding Cas6-3) (Figure 1). Inspection of the RNA sequence reveals a repeat-like sequence with significant similarity (nucleotides in grey) to the AB-repeat family upstream of the mature 5′-end of ncRNA60 (Figure 6). A synthetic version of the ncRNA60 precursor is cleaved by Cas6-3 with similar efficiency to that observed for repeats of the AB or CD families (Figure 6), generating mature ncRNA60 with a 5′ sequence that closely resembles the 5′-handle of crRNAs. This suggests that the ncRNA60 precursor is cleaved by Cas6-3 and loaded into the CSM complex. The processed ncRNA60 sequence is predicted by mfold (28) to form a hairpin structure with an 8 bp helical stem. The functional significance of these observations are unclear at present. It is likely that this sequence does not direct cleavage of the cognate genomic locus as, like the CRISPR loci themselves, there will be no mismatch present at the junction of the 5′-handle and the spacer sequence.

## DISCUSSION

### Co-evolution of Cas6 enzymes with effector complexes and CRISPR repeats

A recent bioinformatics analysis of archaeal CRISPR-Cas systems has revealed that many species encode more than one effector complex, often with only a single *cas6* gene (29). The *S. solfataricus* CRISPR-Cas system is one of the most complex characterized to date. The genome encodes at least five different surveillance complexes of three sub-types, two CRISPR-repeat families and five Cas6 paralogues (12). In situations where multiple CRISPR-repeat families and Cas6 paralogues co-exist, one possibility is that pre-crRNA from any repeat family is processed in a promiscuous manner by the first Cas6 to encounter them. This appears to be the situation in the archaeon *Methanosarcina mazei* where there are two effector complexes (of subtypes I-B and III-B), two Cas6 paralogues and two different repeat sequences, as no differential specificity was observed for either Cas6 with respect to the two repeat sequences (30). Similarly, the two Cas6 paralogues in *Thermus thermophilus* appear to have similar CRISPR RNA cleavage specificities, even though there are distinct modes of RNA recognition (31). The alternative is a tight co-evolution of a Cas6 enzyme specific for its cognate CRISPR-repeat. This appears to be the case in the cyanobacterium *Synechocystis* sp. PCC6803, which has three spacer loci, each associated with genes encoding an effector complex (one type I-D and two type III). Two of these loci have an associated *cas6* paralogue and genetic analysis suggests each Cas6 is specific for its cognate CRISPR-repeat sequence (32), suggesting co-evolution of each Cas6 with its cognate repeat sequence, although it should be emphasized that biochemical data in support of this finding is not yet available.

Our data suggest a more subtle situation exists in *S. solfataricus*. Two CRISPR-repeat families have been described, both capable of forming a 3-bp hairpin stem that is known to provide an important recognition motif for the Cas6-1 family (15) and both generating an identical eight nucleotide 5′-handle sequence upon cleavage by Cas6. The main difference between the AB and CD families occurs in the hairpin loop sequence where three nucleotides out of five vary. This could provide opportunities for differential recognition on binding by different Cas6 paralogues. Cas6-1 is clearly highly specific for the CD family repeat sequence, probably due to the specific recognition of the hairpin loop sequence observed in the co-crystal structure (15). In contrast, the Cas6-3 enzyme has a more relaxed specificity, at least *in vitro*, cleaving all repeat sequences with similar kinetics. The difference in specificities is exemplified by the finding that an abundant non-coding RNA species in *S. solfataricus*, ncRNA60, is generated from a precursor molecule by cleavage mediated by Cas6-3 but is not a substrate for Cas6-1. Closer inspection of the RNA precursor reveals extensive sequence identity with the AB-family CRISPR-repeat sequence (Figure 6C) including all five nucleotides of the hairpin loop and the sequence flanking the Cas6-3 cleavage site. However, tellingly, the ncRNA60 precursor lacks the ability to form a hairpin stem structure, with only one out of three base pairs possible. This raises the possibility that the Cas6-3 family, for which there is no structural information, utilize a fundamentally different mechanism for repeat recognition that does not rely on cleavage at the base of a hairpin. Although hairpin loops are a recurring feature in Cas6:repeat complexes the structure of *P. furiosus* Cas6 bound to a repeat RNA suggests an alternative ‘wrap around’ recognition mechanism is possible (18). Further studies are required to explore this possibility.

### Coupling Cas6 to effector complexes

The established paradigm for Cas6 enzymes is that they catalyse only a single turnover, remaining tightly bound to the crRNA cleavage product with picomolar affinities (25,31,33). This is logical where Cas6 is an integral subunit of the type I Cascade complex or where a dedicated Cas6 is present for each effector complex, a situation that is possible, at least in theory, in *S. solfataricus*. Alternatively, there could be a complete disconnect between Cas6 and the effector complexes, with crRNAs dissociating from Cas6 enzymes following cleavage, leaving them free to associate with effector complexes stochastically. The reality appears to lie somewhere between these extreme scenarios.

On the one hand, Cas6-1 is unique amongst currently characterized Cas6 enzymes in its ability to sustain multiple turnover catalysis—acting as a true enzyme and generating free crRNA product *in vitro*. The kinetic constants suggest that product release does not significantly limit the overall rate of reaction, unlike other Cas6 enzymes studied in detail (25,31). This may be largely due to weaker binding of substrates and products by Cas6-1, where dissociation constants are about 15 nM rather than the picomolar *K*_D_'s observed for other enzymes. In this scenario, crRNAs processed by Cas6 could be released to act as a focal point for the assembly of effector complexes of types IA, III-A and III-B in *S. solfataricus*. This is consistent with the observation that no Cas6 paralogue is found in association with purified type III complexes in *S. solfataricus* or *P. furiosus* (19,20,34). Furthermore, in the related species *S. islandicus* REY15A, one Cas6 appears to service the crRNA requirements of three different effector complexes (one type I-A and two type III-B) (35).

**Figure 7.**
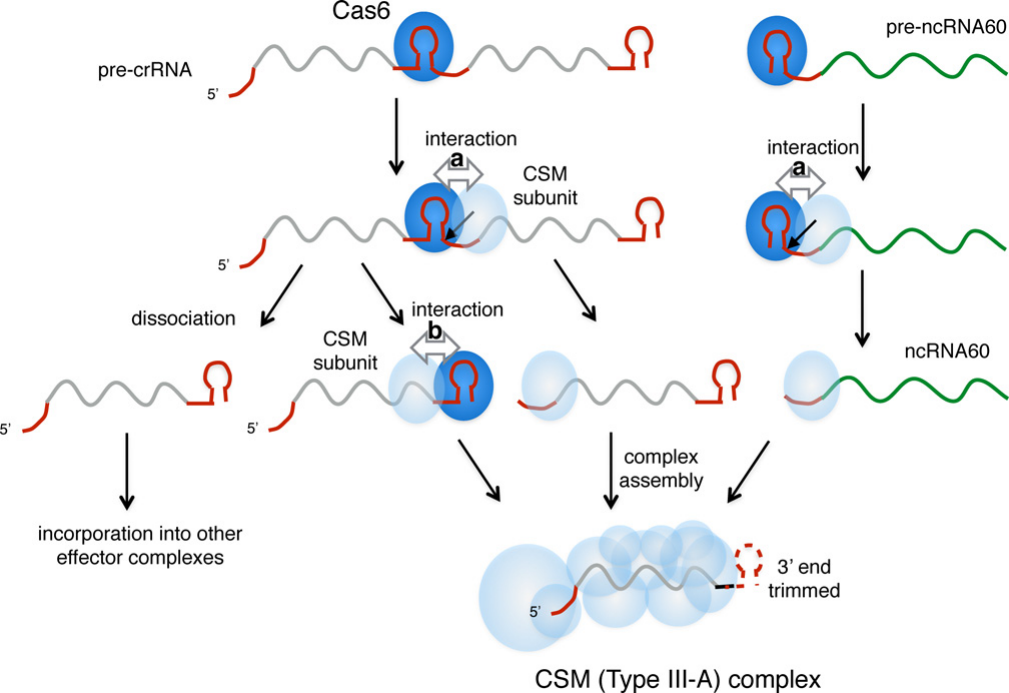
Model for biased uptake of AB-family crRNAs into the CSM complex. Cas6 binding to pre-crRNA creates two possibilities for interactions between bound Cas6 and CSM subunits: (**a**) 3′ of the cleavage site at the nascent 5′-handle sequence, or (**b**) 5′ of the cleavage site, either before or after cleavage by Cas6. Either alternative could explain the enrichment of AB family crRNAs in the CSM complex due to an interaction between a CSM subunit and Cas6-3. A third possibility is the dissociation of Cas6 leaving free crRNA to initiate assembly of effector complexes. A similar mechanism could account for biased uptake of ncRNA60 into the CSM complex via interaction a) only (shown on right). Spacer sequence is shown in grey and repeat-derived sequences in a darker shade.

On the other hand, there is clearly a biased uptake of crRNAs in the CSM complex, with 88% derived from the A and B loci (20). Coupled with the observation that the ncRNA60 species is also present at high levels in CSM, this suggests a functional link between Cas6-3 (and/or Cas6-2, which has not yet been characterized) and the CSM complex. In the CMR complex, the situation is reversed with a biased uptake of crRNA from loci C and D (19). It should be borne in mind that mature crRNAs from the AB and CD loci are indistinguishable from one another at the 5′-end where the 5′-handle sequences are identical. This suggests some type of handover between Cas6 and the downstream effector complexes may be operating. It is possible that Cas6 binding to repeat sequences facilitates the initiation of CSM complex assembly with crRNA via protein:protein interactions. These crRNA binding events could occur either 5′ or 3′ of the bound Cas6 (Figure 7). Transient interactions between Cas6 proteins and the type III complexes could be lost when crRNA product dissociates from Cas6 or when the remaining repeat-derived RNA of the 3′-handle is trimmed away—a feature of type III systems (5,19,20,36). It should be noted that the ncRNA60 precursor has only a single ‘repeat-like’ motif and any interaction between CSM subunits and Cas6-3 on this RNA species would have to occur near the 5′-end prior to cleavage by Cas6-3. This interaction raises the possibility that different effector complexes might target different types of mobile element, depending on the origins of spacers found in the different CRISPR loci.

## CONCLUSION

In conclusion, we have studied crRNA processing and loading into effector complexes in the highly complex *S. solfataricus* CRISPR-Cas system. Two families of Cas6 proteins have been investigated, revealing differing specificities for the CRISPR-repeat sequences present and the possibility of multiple turnover catalysis to generate free crRNA. Biased incorporation of AB family repeats into the CSM complex may be explained by transient interactions with Cas6:crRNA product complexes. We have also shown that Cas6 enzymes can process non-crRNA *in vivo*, suggesting alternative potential functions for these enzymes beyond the CRISPR-Cas system.
